# Travel for survive! Identifying the antecedents of vaccine tourists' travel intention: Using a stimulus-organism-response model

**DOI:** 10.3389/fpubh.2022.850154

**Published:** 2022-07-29

**Authors:** Xue-Bing Wang, Chien-Chao Chen, Gordon Chih Ming Ku, Che-Hsiu Chen, Chin Hsien Hsu, Peng-Yeh Lee

**Affiliations:** ^1^School of Tourism Management, Wuhan Business University, Wuhan, China; ^2^Department of Leisure and Recreation Management, Asia University, Taichung, Taiwan; ^3^Department of Sport Management, National Taiwan University of Sport, Taichung, Taiwan; ^4^Department of Sport Performance, National Taiwan University of Sport, Taichung, Taiwan; ^5^Department of Leisure Industry Management, National Chin-Yi University of Technology, Taichung, Taiwan; ^6^Department of Business Administration, National Yunlin University of Science and Technology, Douliu, Taiwan

**Keywords:** medical tourism, vaccine tourism, risk perception, pandemic prevention attitude, travel decision making, travel intention, COVID-19

## Abstract

The COVID-19 global pandemic and the uneven distribution of vaccines have resulted in alternative medical tourism, vaccine tourism. The purpose of this study is to identify the antecedents of vaccine tourists' travel intention. The Stimulus-organism-response model was used as a framework to understand the relationship between risk perception (stimulus), pandemic prevention attitude (organism), decision making (organism), and travel intention (response) in vaccine tourism. An online questionnaire survey method was adopted to address the purpose of the research. Purposive and snowball sampling were used to select eligible respondents who were over 18 years old and had experience in vaccine tourism. A total of 520 online questionnaires were collected, and description analysis, confirmatory factor analysis, and structural equation modeling were utilized to analyze the collected data. The findings indicated that pandemic prevention attitude is a full mediator between risk perception and travel intention. There is a significant causal relationship between risk perception and pandemic prevention attitude and between pandemic prevention attitude and travel intention. Furthermore, tourists' travel decision-making also significantly influences their travel intention. However, the relationship between tourists' risk perception and travel decision-making has no significant effect. Vaccine tourism was created based on the COVID-19 context. Therefore, in order to avoid vaccine travel becoming an infection control breach, pandemic prevention planning and the medical quality of the destination, and the prevention policies between the countries should be completely assessed and conducted.

## Introduction

### Background and problem statement

Medical tourism is an international or long-distance tourism activity that aims to obtain medical services and combines leisure, business, and other purposes (1, 2). In the COVID-19 context, the global pandemic and the uneven distribution of vaccines have resulted in the prevalence of alternative medical tourism, vaccine tourism. Taiwan faces the problem of vaccine shortage during the pandemic, which has led to people traveling abroad for vaccination. According to statistics from the Tourism Bureau (3), since the United States opened the vaccine system for foreign tourists, many Taiwanese tourists to the United States have increased every month. For example, in May 2021, the number of Taiwan tourists to the United States reached 7,188, an increase of 428.53% over the same period last year. Thus, it can be seen that, even though there are certain risks in going abroad for vaccination, the uncertainty of pandemic control and vaccine supply is one of the main reasons for Taiwanese people to travel abroad for vaccination.

Tourism risk perception is one issue that influences tourists' attitude and decision-making process toward tourism, which hinders tourists' travel intentions (4, 5). Tourists usually assess the risks they may encounter during their travel, including the risks between the origin and destination and the destination itself, and determine their travel intention or change the destination according to the perceived risks (6, 7). Zhu and Deng (8) found that, during the COVID-19 pandemic, there has been a significant negative relationship between the risk perception and travel intention of rural tourism tourists. However, the journey, especially during aircraft travel, is one of the common ways to be infected with COVID-19 (9), and while the control of the pandemic abroad is not explicit, Taiwanese still choose to go abroad to get vaccinated. Therefore, whether the relationship between risk perception, attitude, and decision-making regarding vaccine tourism in the COVID-19 context is different from that of other tourism activities is one of the focuses of this study.

The pandemic prevention attitude is an essential factor directly affecting tourists' travel intention in the COVID-19 context (4, 5). The pandemic prevention attitude of tourists represents their confidence in pandemic control, which is related to the integrity and implementation of pandemic prevention measures taken by government departments (10). The more positive the pandemic prevention attitude of tourists, the higher their travel intention (4). However, the relationship between pandemic prevention attitude and travel intention may vary with different tourism activities. In terms of vaccine tourism in Taiwan, most Taiwanese tourists traveling abroad for vaccination will consider the uncontrolled pandemic situation and lack of access to vaccines locally, which has indirectly contributed to the development of vaccine tourism and is different from other tourism situations. Therefore, the relationship between the pandemic prevention attitude of vaccine tourists and their travel intention is worthy of further study.

Consumer behavior, decision-making factors are essential mechanisms affecting tourists' behavior (11, 12). The decision-making process of tourists is influenced by tourists' demand cognition, information search, scheme evaluation, consumption behavior, post-consumption evaluation, and feedback (13). Therefore, tourists' travel attitudes, activities, ideas, and experiences are all factors that affect their travel decision-making process (14). Many previous studies have shown that the factors influencing tourists' decision making have a significant impact on their choice of tourist destinations and their behaviors (15), and the research topics were mainly mass tourism, outbound tourism (16), domestic tourism (17), marine tourism (18), and medical tourism (19–21). Although many studies focused on exploring the factors influencing the decision-making of medical tourism, most types of medical tourism were mainly for health care, such as health examinations. Therefore, they were usually under the condition that they were allowed to travel abroad usually. In the context of COVID-19, vaccine tourism is another way to ensure life safety for countries with a shortage of vaccines, rather than promoting physical health. Moreover, when traveling abroad for vaccine tourism, it is no longer enough just to consider the quality of local medical services, reputation, and destination image, meaning the personal pandemic prevention attitude and risk of infection are the priority factors affecting tourists' travel decisions (22) when determining whether or not to travel abroad for vaccination.

A stimulus-organism-response (SOR) model can be seen as a framework for exploring the vaccine tourism behavior in the context of COVID-19. The SOR model has been used to measure the link between the input (stimulus), process (organism), and output (response) of the tourism behavior and to understand the factors and processes that influence tourists' behavior (23, 24). In retrospect, the related topics of SOR model application in tourism include virtual reality tourism (23), honeymoon tourism (25), nature-based tourism (26), and sports tourism (27). These studies found a variety of different stimulus variables and process variables to predict tourist behavior. Their results provide an effective way to further understand the behavior of tourists during different types of tourism; however, no studies are using the SOR model to understand tourists' behavior in vaccine tourism. Especially during the global COVID-19 pandemic, the risk perception of tourists (stimulus) may affect their pandemic prevention attitude (organism), which in turn affects their decision making to travel abroad for vaccination, and finally, leads to their behavioral intention of vaccine tourism (response). Accordingly, in this study, the SOR model was used as the framework to explore the relationship between the risk perception, pandemic prevention attitude, decision making, and travel intention of vaccine tourists traveling abroad for vaccination.

### Stimulus-organism-response model

The SOR model is proposed by Mehrabian and Russell (28) and is used to understand the impact of external environmental stimulus (S) on individual emotions (O), and finally, form specific behaviors (R). In tourism, the SOR model is used to explain the impact of a tourist destination environment on tourists' inner emotions and the generation of their behaviors (26). The SOR model is composed of antecedent variables, intervening variables, and outcome variables (28). The antecedent variable refers to the external environment attributes. Tourists will stimulate their psychological state through their five senses. The intervening variables refer to the process of the internal psychological responses produced by external stimuli, representing tourists' emotional state. The outcome variables refer to the behavioral responses of tourists, which emotions may influence to produce the approach behavior and avoidance behavior (28). From the perspective of consumer behavior, the approach behavior refers to the positive behavioral response of consumers caused by the stimuli of the external environment, which can be regarded as consumers' desire to stay, look around, explore, and socialize in a specific environment (29). On the contrary, avoidance behavior refers to the negative behavioral response produced by consumers after receiving environmental stimuli, and their intention to stay in the environment is relatively low (29). Among them, the approach behavior can be divided into actual approach behavior and intended approach behavior; the actual approach behavior refers to the behavior that has been realized, while the intended approach behavior refers to the intention of the behavior (30, 31). In tourism research, travel intention is usually used to measure the intended approach behavior of tourists (23). According to the SOR model, this study set the risk of tourists to the external environment of tourism as an antecedent variable (S), which may have an impact on their pandemic prevention attitude (O1) and decision-making process (O2). It will ultimately determine the intention to travel abroad for vaccination (R).

### Stimulus: Risk perception

Risk perception refers to the possibility of negative results (32). Tourism risk perception can be regarded as the negative result or process that tourists' subjective judgment may produce in the tourism environment, mainly due to the asymmetry between tourism safety information and subjective perception (8, 33). Tourism risk perception is based on tourists' environmental knowledge of the destination and generated through subjective and objective assessment, beyond the critical point of negative impact in the journey (24). Therefore, tourism risk perception can be regarded as the risk awareness generated by external environmental stimuli in tourism. However, the risk perception of vaccine tourism is different from that of other types of tourism activities, and there is a certain degree of risk in treatment. Therefore, in addition to medical treatment and tourism costs for tourists, the risk of nosocomial infection caused by different sanitary conditions is also one of the major risks of medical tourism in other countries (34, 35). Similarly, in addition to the high cost of travel, medical treatment, and quarantine when traveling abroad for vaccination, the most critical risk of vaccine tourism is the possibility of catching the disease. However, due to the shortage of vaccines in Taiwan, even if the pandemic situation in foreign countries has not been controlled, people are still traveling abroad for vaccination to protect their lives. Therefore, it is worth exploring whether tourism risk perception will affect tourists' intention to medical tourism, as well as their pandemic prevention attitude and travel decision-making.

Tourists' attitude is related to their risk perception in the COVID-19 context (5). As risk perception represents a loss to some extent, it has a negative impact on tourists' behavior and attitude (36); for example, Rather (5) found that tourists' risk perception had a significant negative impact on their attitude. However, a study on untact travel by Bae and Chang (36) found that tourism risk perception had a significant positive impact on attitude, as they considered untact travel as a health promotion behavior to reduce tourism risk. Similarly, vaccination is also a health promotion behavior; the higher tourists' risk perception, the more positive their pandemic prevention attitude. Accordingly, this study proposes the following hypothesis:

H1: Vaccine tourists' risk perception significantly influences their pandemic prevention attitude.

The risk perception of tourists has a significant impact on their travel decision-making (37). When tourists believe that tourism satisfaction will be reduced due to risk perception, they usually change the process of travel decision making and the choice of destination (7); the higher the risk perception, the higher the chance to change the decision. Polas et al. (37) found that the risk perception of medical tourism had a significant positive impact on travel decision-making. Therefore, the travel decision-making process of vaccine tourists may also be affected by risk perception. Thus, this study proposes the following hypothesis:

H2: Vaccine tourists' risk perception significantly influences their travel decision-making.

High-risk perception is the main reason for reducing tourists' travel intention (38). Although many studies have explored the relationship between tourists' risk perception and travel intention, the research results showed that the relationship between the two variables was not very stable. For example, Qi et al. (39) found no significant correlation between the perception of financial risk, physical risk, psychological risk, social risk, and time risk of mega-event tourists and their travel intention. On the contrary, in their research on tourism after the nuclear disaster in Japan, Chew and Jahari (38) found that financial risk, physical risk, psychological risk, and social risk all had a significant negative impact on travel intention; therefore, the relationship between risk perception and travel intention will vary according to the different types of tourism activities. The relationship between the risk perception and travel intention of vaccine tourists has not been explored, and it is worth further understanding, especially in the context of COVID-19. Based on the above literature review, this study proposes the following hypothesis:

H3: Vaccine tourists' risk perception significantly influences their travel intention.

### Organism: Pandemic prevention attitude and decision-making

The pandemic prevention attitude and travel decision-making can be regarded as the psychological state generated by tourists' perception of the external physical risking the COVID-19 context. The pandemic prevention attitude refers to people's perception of the decisions or beliefs about implementing pandemic prevention policies (4). The perception of the pandemic prevention attitude is based on the effectiveness of the government in controlling the pandemic, which affects people's confidence in participating in tourism activities (3). Luan et al. (40) suggested that pandemic prevention strategies can be divided into primary and secondary strategies. The primary strategy is a direct pandemic prevention method, meaning vaccination. The second strategy is indirect pandemic prevention, including wearing masks, isolation from infected persons and close contacts, regular disinfection of public places, mass screening, and strengthening public awareness health education. Effective pandemic prevention strategies and knowledge advocacy can help to increase civic behavior among tourists, ensure travel safety, and strengthen social forces against the COVID-19 pandemic (41). Tseng et al. (4) found that the pandemic prevention attitude of tourists had a significant positive impact on their travel intention; therefore, the more positive the pandemic prevention attitude of vaccine tourists, the higher their intention to travel abroad for vaccination. Accordingly, this study proposes the following two hypotheses:

H4: Vaccine tourists' epidemic prevention significantly influences their travel intention.
H5: Vaccine tourists' risk perception indirectly influences travel intention through pandemic prevention attitude.

Travel decision-making is a factor that directly affects tourists' travel intention. Travel intention can be regarded as the result of tourists' psychological decision-making process, which leads to the actual tourism behavior and transforms perceived value into actual behavior (42). Risk perception is one of the critical factors affecting the decision-making process of tourists. Especially in the COVID-19 context, understanding the relationship between tourists' tourism risk perception and travel decision-making can help the tourism industry draw up restart and recovery plants in the post-pandemic era and provide clear supply management strategies for the tourism industry (42). Many studies have confirmed a significant relationship between risk perception, travel decision-making, and travel intention (42–44). This study focused on the relationship between tourism risk perception, decision making, and travel intention of vaccine tourists. Accordingly, this study proposes the following two hypotheses:

H6: Vaccine tourists' travel decision-making significant influences their travel intention.
H7: Vaccine tourists' risk perception indirectly influences travel intention *via* travel decision-making.

### Response: Travel intention

**Figure 1:**
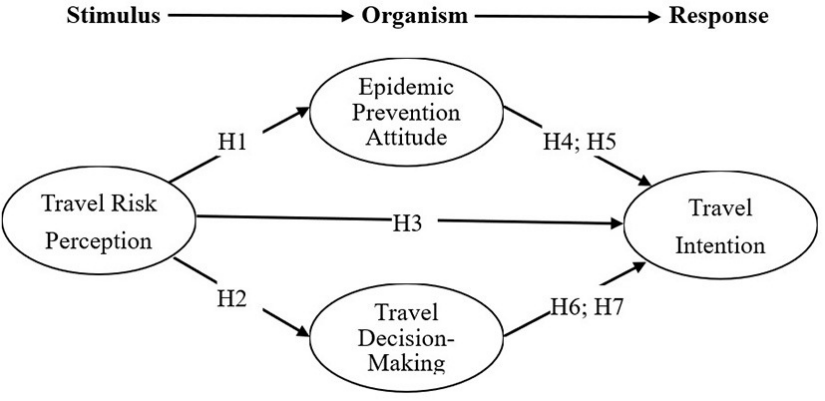
Research framework of medical tourists' intention.

Travel intention is the intention of tourists to transform the results and motivation of the psychological process into actual behavior, which can also be regarded as a form of behavior intention (45). Travel intention is generated by rationally evaluating the potential benefits and costs of a series of alternative tourist destinations through external information (46). Thus, medical tourism intention can be regarded as tourists' rational evaluation of medical and tourism information, and then, they transfer the decision-making and motivation into the intention of actual behavior. A review of relevant studies on the travel intention of medical tourism shows that the factors affecting travel intention include electronic word-of-mouth (47, 48), destination trust (47), motivation (49), risk perception (49), destination image (48–50), attitude (50), subjective norms (50), and perceived behavioral control (50). However, in the COVID-19 context, there is a lack of research on tourists' risk perception, pandemic prevention attitude, travel decision making, and travel intention in medical tourism with vaccination as the main purpose, especially in countries with a shortage of vaccines. Based on the above literature review and the SOR model framework, this study proposes the following research framework (Figure 1).

## Methods

### Research design

This study used the SOR model to explore the relationship between tourism risk perception, pandemic prevention attitude, travel decision-making, and travel intention of vaccine tourism among Taiwanese people traveling abroad for vaccination. After determining the research topic, a quantitative research method was selected as the basis of the overall research design to meet the research issue. This study collected data through a questionnaire survey, and the questionnaire was prepared by referring to relevant literature. Before the formal distribution of the questionnaire, this study adopted a pre-test and invited two experts in related fields to review the questionnaire content to ensure its surface validity and content validity. Furthermore, this study selected adults over 18 years of age as the subjects for the formal distribution of the questionnaire, and questionnaire data were collected through online questionnaires. Finally, statistical software was used to analyze the data and present the results.

### Data collection

This study used purposive and snowball sampling to select tourists over 18 who had traveled abroad for vaccination as the research objects. Tourists under the age of 18 and those who had not traveled abroad for vaccination were excluded. This study used google forms as the platform for questionnaire data collection (https://docs.google.com/forms). Eligible respondents were invited to fill out the questionnaire through online social network platforms, Line and Facebook, and were asked to share the online questionnaire with their acquaintances. Snowball sampling assisted this study in reaching the respondents who were impossible or prohibitively expensive (51). Especially in the COVID-19 context, social distance is a huge constraint for investigation. Snowball sampling increased the efficiency and quality of questionnaire distribution. The questionnaires were collected from July 1 to July 31, 2021. During this period, five hundred and fifty online questionnaires were collected, and 520 valid questionnaires were obtained after excluding the invalid questionnaires (the answers were all the same and the IP addresses of the online questionnaires were repeated) for a valid questionnaire rate of 94.5%.

### Measures

The questionnaire of this study consists of three parts. The first part is the content of informed consent, including the description of the purpose of this study and the related rights of the research objects. The second part is the scale of tourism risk perception, pandemic prevention attitude, travel decision-making, and travel intention (Table 1). The tourism risk perception (17) included three dimensions, namely “social risk”, “psychological risk”, and” financial risk”, with seven items in total; the pandemic prevention attitude scale has consisted of three items. The travel decision-making scale (17) contained three dimensions, namely “commercial source”, “experiential source”, and “public source”, with six items in total; Travel intention scale was comprised of three items. A Likert 5-point scale was used to be the measurement benchmark, with “Strongly agree” to “Strongly disagree” represented by 5 to 1 points, respectively. The third part is the demographic, including gender, age, marriage, education level, occupation, average monthly income, and residence.

**Table 1:** Scale of tourism risk perception, pandemic prevention attitude, travel decision-making, and travel intention.

**Variables**	**Dimensions**	**Items**
Tourism risk perception	Social risk	Worried about the poor attitude of local medical staff (X1)
		Worried about the unfriendly treatment by local people (X2)
	Psychological risk	Fear of complications caused by the COVID-19 vaccine (X3)
		Concerns about medical errors or disputes arising from the COVID-19 vaccination (X4)
	Financial risk	Fear that COVID-19 vaccine is not as effective as expected and wastes money (X5)
		Concerns about costly travel to local areas for COVID-19 vaccination (X6)
Pandemic prevention attitude	–	It is wise to take precautions against COVID-19 in advance (X7)
		It is correct to take precautions against COVID-19 in advance (X8)
		For me, it is important to take precautions against COVID-19 in advance (X9)
Travel decision making	Commercial source	Advanced medical equipment for COVID-19 vaccination in foreign destinations (X10)
		High level of medical services for COVID-19 vaccination in foreign destinations (X11)
	Experiential source	Comfortable environment of hotels in foreign destinations (X12)
		Beautiful scenic spots in foreign destinations (X13)
	Public source	Complete/perfect insurance system in foreign destinations (X14)
		Complete/perfect legal protection in foreign destinations (X15)
Travel intention	–	I think overseas vaccine tourism is worthwhile (X16)
		If the budget permits, I will travel abroad for vaccination (X17)
		When the COVID-19 vaccine is not available in Taiwan, I will choose overseas vaccine tourism (X18)


### Ethical considerations

In order to comply with research ethics, an anonymous questionnaire survey was conducted in this study to ensure that the personal information of respondents could not be identified. The research respondents must be at least 18 years old to participate in this study, and those under 18 years old were excluded. This study provided the content of informed consent on the first page of the questionnaire to inform the purpose of the study and the respondents' rights. The respondents understood and agreed to participate in the survey voluntarily. If the respondents were not willing to participate in the survey or withdrew from this study during the completion, their rights and interests would not be affected, and the results would not be adverse. The collected data can only be reviewed and analyzed by the researchers and will not be used for any purpose other than academic research. The researchers will destroy the data themselves after the publication of this study.

### Data analysis

This study used SPSS18.0statistical software to analyze the mean, standard deviation, skew, and kurtosis of the demographic background variables and questions. AMOS 23.0 structural equation was used to conduct confirmatory factor analysis (CFA) to test the reliability and validity of the variables in this study. Structural model analysis was used to test the relationship between tourism risk perception and pandemic prevention attitude, travel decision making, and travel intention.

## Results and discussion

### Demographic

According to the demographic of the samples in this study, men (55.0%) were slightly more than women (45.0%). Most of the samples were aged from 41 to 50 (29.8%). More than half of the samples were married (68.2%). Nearly half of the samples earned more than NTD 45001 per month on average (45.6%) 0.55.6% of the samples had a college or junior college degree. Most of the samples lived in the central part of Taiwan (68.8%). The samples engaged in the service industry (45.2%) were the largest in terms of occupation (Table 2).

**Table 2:** Respondents' demographics (*n* = 520).

**Variables**	**Frequency**	**Percentage (%)**	**Variables**	**Frequency**	**Percentage (%)**
Gender			Age		
Men	286	55.0	18~20 years old	8	1.5
Women	234	45.0	21~30 years old	43	8.3
Marriage			31~40 years old	137	26.3
Married	335	68.2	41~50 years old	155	29.8
Single	148	28.5	51~60 years old	147	28.3
Others	17	3.3	Over 61 years old	30	5.8
Average monthly income (NTD)			Education LEVEL		
Under 20,000	38	7.3	Junior high	4	0.8
20,001~25,000	31	6.0	Senior high	82	15.8
25,001~30,000	53	10.2	Undergraduate	289	55.6
30,001~35,000	48	9.2	Graduate	145	27.8
35,001~4,000	65	12.5	Occupation		
40,001~45,000	48	9.2	Student	25	4.8
Over 45,001	237	45.6	Government	96	18.5
Residence			Service	235	45.2
Northern *Taiwan*	77	14.8	Machinery	6	1.2
Central *Taiwan*	358	68.8	Business	30	5.8
Southern Taiwan	68	13.1	Technology	10	1.9
Eastern Taiwan	5	1.0	Healthcare	13	2.5
Offshore Islands	12	2.3	Freelancer	73	14.0
			Others	32	6.2


### Confirmatory factor analysis

Confirmatory factor analysis was used to test the reliability and validity of tourism risk perception, pandemic prevention attitude, travel decision making, and travel intention scale. In order to avoid affecting the estimation and test results of the structural equation model, the sample data should be verified before the analysis. The absolute kurtosis values of the observed variables should be <10, and the absolute skew values should be <3. If the observed variables meet the above conditions, the maximum likelihood method can be used for model estimation (52). As can be seen from Table 3, tourism risk perception (skew: −0.173 to −0.803; kurtosis: −0.120 to −1.084), pandemic prevention attitude (skew: −0.601 to −0.918; kurtosis:-0.337 to 0.743), travel decision making (skew: −0.019 to −0.520; kurtosis: 0.002 to 0.964), and travel intention (skew: −0.433 to −0.650; kurtosis: −0.102 to 0.178), the absolute skew values of all observed variables are <3, and the absolute kurtosis values are <10. Thus, the structural equation in this study is suitable for analysis by the maximum likelihood method.

**Table 3:** Analysis of mean, standard deviation, skew, and kurtosis of variables.

**Construct**	**Variable**	**Mean**	**Standard deviation**	**Skewness**	**Kurtosis**
Tourism risk perception	X1	3.713	1.154	−0.803	−0.120
	X2	3.559	1.149	−0.631	−0.480
	X3	3.463	1.335	−0.428	−1.064
	X4	3.738	1.080	−0.623	−0.155
	X5	3.634	1.179	−0.533	−0.654
	X6	3.348	1.227	−0.173	−1.084
Pandemic prevention attitude	X7	4.236	0.828	−0.730	−0.337
	X8	4.153	0.879	−0.918	0.743
	X9	4.146	0.854	−0.601	−0.521
Travel decision making	X10	3.505	0.903	−0.277	0.212
	X11	3.369	0.952	−0.166	0.002
	X12	3.613	0.859	−0.520	0.964
	X13	3.703	0.826	−0.513	0.830
	X14	3.151	0.948	−0.019	0.047
	X15	3.221	0.990	−0.121	0.052
Travel intention	X16	3.728	0.988	−0.650	0.178
	X17	3.792	0.896	−0.464	−0.105
	X18	3.775	0.915	−0.433	−0.102


Before analyzing the model fit, to avoid improper explanation, it is necessary to examine whether the measurement model has offending estimates or the parameter estimate exceeds the acceptable range. General offending estimates occur with negative standard errors, standardization coefficients exceeding or too close to 1 (usually 0.95 as the critical point), and substantial standard errors (52). As can be seen from the analysis results of model parameter estimation (Table 4), the standard error of the observed variables in this study ranges from 0.04 to 0.10, meaning without substantial standard error and negative value. In contrast, the standardization coefficient ranges from 0.65 to 0.95 without any standardization coefficient exceeding or approaching 1. Therefore, the measurement model in this study has no offending estimate and is suitable for the fit analysis of the measurement model.

**Table 4:** Model parameter estimation table of variables.

**Pointer/Construct**	**Standardized regression coefficient**	**Unstandardized regression coefficient**	**(SE)**	**(C.R.)**
X1 < -Social risk	0.647	1.000		
X2 < -Social risk	0.873	1.344	0.095	14.126
X3 < -Psychological risk	0.948	1.000		
X4 < -Psychological risk	0.693	0.592	0.039	15.274
X5 < -Financial risk	0.898	1.000		
X6 < -Financial risk	0.658	0.762	0.055	13.773
X7 < -Pandemic prevention attitude	0.874	1.000		
X8 < -Pandemic prevention attitude	0.895	1.088	0.045	24.066
X9 < -Pandemic prevention attitude	0.778	0.918	0.044	20.964
X10 < -Commercial source	0.694	1.000		
X11 < -Commercial source	0.902	1.371	0.097	14.090
X12 < -Experiential source	0.922	1.000		
X13 < -Experiential source	0.663	0.692	0.048	14.367
X14 < -Public source	0.830	1.000		
X15 < -Public source	0.829	1.044	0.054	19.252
X16 < -Intention	0.628	1.000		
X17 < -Intention	0.891	1.287	0.087	14.741
X18 < -Intention	0.872	1.285	0.085	15.162

In this study, absolute and relative fit measures were used to measure the model fit of the measurement and structural models. The absolute fit measures include GFI, AGFI, and RMSEA (52), while the relative fit measures include NFI, TLI (NNFI), CFI, IFI, and RFI. The results of measurement model fit show that the absolute fit measures, meaning GFI (0.94) and AGFI (0.91), are both >0.90, and RMSEA was 0.06, while the relative fit measures, NFI (0.94), TLI (NNFI) (0.94), CFI (0.96), IFI (0.96), and RFI (0.91) are all >0.90, indicating that the measurement model in this study has a reasonable degree of fit (Table 5).

**Table 5:** CFA model.

**Model fit indices**	**Measurement values**
Absolute Fit Indices	GFI	0.941
	AGFI	0.906
	RMSEA	0.058
Incremental fit indices	NFI	0.939
(Comparative fit indices)	TLI/NNFI	0.943
	CFI	0.960
	IFI	0.960
	RFI	0.912

**Figure 2:**
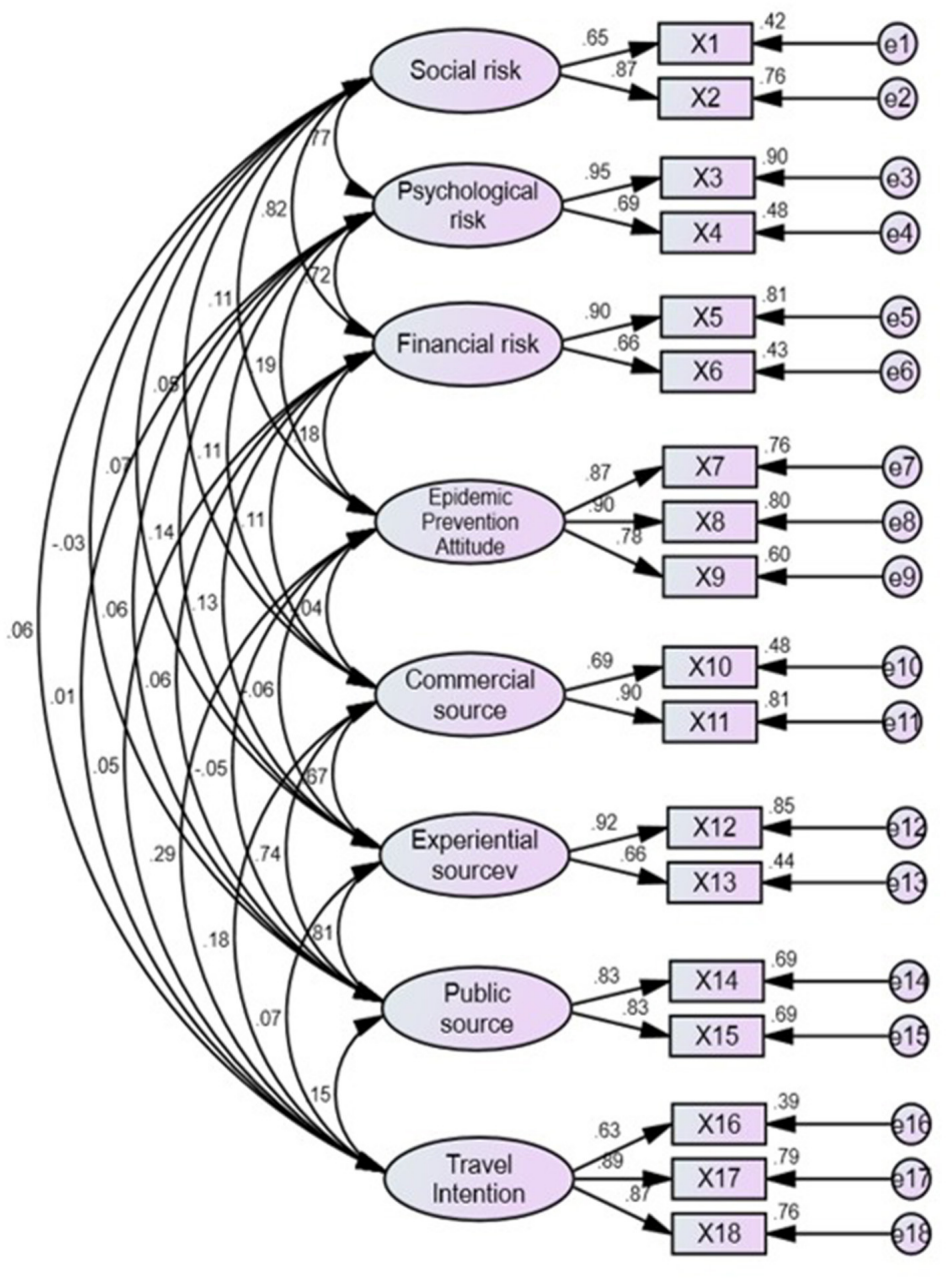
CFA model (Model fit indices: GFI = 0.941, AGFI = 0.906, RMSEA = 0.058, TLI/NNFI = 0.943, CFI = 0.960, IFI = 0.960, RFI = 0.912).

In terms of the reliability and validity of the scale (Figure 2, Tables 6, 7), Cronbach's α, the reliability of individual observed variables, construction reliability, mean extraction variation, aggregation validity, and discriminant validity were used as the basis for measurement (52). The Cronbach's coefficients of all potential variables exceeded 0.70, indicating the good internal consistency of potential variables. The R2 coefficients (variation ratio) of the reliability of individual observed variables were all >0.20, indicating the good internal consistency of individual observed variables. The construction reliability coefficients were all >0.60, indicating a high correlation between the observed variables. The mean extraction variation was above 0.50, indicating that more than half of the explanatory variables of the dimensions were from the observed variables themselves. The factor loads of the observed variables were all higher than 0.50, indicating that the observed variables could effectively reflect the potential variables. The AVE square root coefficients of the discriminant validity of all potential variables were higher than the correlation coefficients between two variables, indicating that each potential variable could be effectively distinguished (52). According to the above findings, the measurement model in this study has good reliability and validity.

**Table 6:** Summary of the convergent validity and construct reliability.

**Variable**	**Facet**	**Variable**	**R^2^**	**Cronbach's α**	**C.R**	**AVE**
Tourism risk perception	Social risk	X1	0.418	0.858	0.738	0.590
		X2	0.763			
	Psychological risk	X3	0.898		0.812	0.689
		X4	0.480			
	Financial risk	X5	0.807		0.760	0.619
		X6	0.433			
Pandemic prevention attitude	-	X7	0.764	0.885	0.834	0.629
		X8	0.802			
		X9	0.605			
Travel decision making	Commercial source	X10	0.482	0.864	0.783	0.647
		X11	0.814			
	Experiential source	X12	0.850		0.779	0.644
		X13	0.440			
	Public source	X14	0.688		0.815	0.688
		X15	0.688			
Travel intention	-	X16	0.395	0.832	0.844	0.649
		X17	0.793			
		X18	0.760			

*C.R, Composite Reliability; AVE, Average Variance Extracted*.

**Table 7:** Discriminant validity.

	**SR**	**PR**	**FR**	**PPA**	**CS**	**ES**	**PS**	**TI**
SR	0.812							
PR	−0.029	0.843						
FR	0.061	0.767	0.742					
PPA	0.062	0.817	0.716	0.849				
CS	−0.054	0.109	0.194	0.182	0.806			
ES	0.736	0.050	0.109	0.114	−0.039	0.800		
PS	0.805	0.066	0.143	0.133	−0.060	0.670	0.825	
TI	0.146	0.058	0.008	0.053	0.292	0.181	0.074	0.800

*SR, Social risk; PR, Psychological risk; FR, Financial risk; PPA, Pandemic prevention attitude; CS, Commercial source; ES, Experiential source; PS, Public source; TI, Travel intention*.

### Structural model analysis

According to the measurement model, reliability and validity in this study reached the standard value. Thus, the structural model analyzed the relationship between tourism risk perception, pandemic prevention attitude, travel decision making, and travel intention. The fit detection results of the structural model show that both the absolute fit measures, GFI (0.933), AGFI (0.907), and RMSEA (0.056), and the relative fit measures, NFI (0.931), TLI (NNFI) (0.945), CFI (0.956), IFI (0.956), and RFI (0.915), meet the standard for model fit, which means that the model obtained in this study has a reasonable degree of fit (Table 8).

**Table 8:** Structural model.

**Model fit indices**	**Measurement values**	
Absolute fit indices	GFI	0.933
	AGFI	0.907
	RMSEA	0.056
Incremental fit indices	NFI	0.931
(Comparative fit indices)	TLI/NNFI	0.945
	CFI	0.956
	IFI	0.956
	RFI	0.915

**Figure 3:**
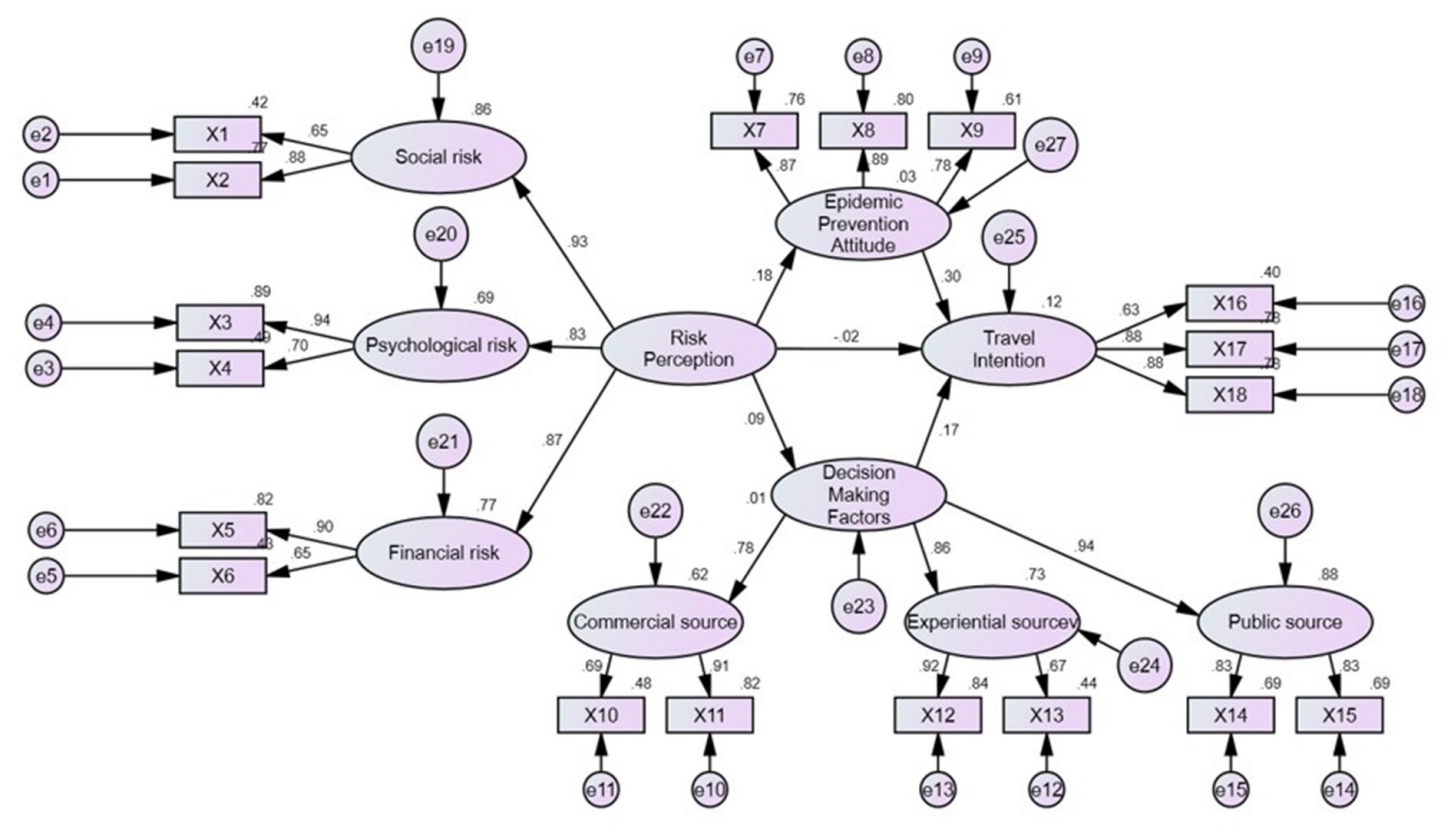
Structural model of the relationship between risk perception, pandemic prevention attitude, travel decision making, and travel intention (Model fit indices: GFI = 0.933, AGFI = 0.907, RMSEA = 0.056, TLI/NNFI = 0.931, CFI = 0.945, IFI = 0.956, RFI = 0.915).

The results of structural model analysis (Figure 3) show that tourism risk perception only has a significant impact on the pandemic prevention attitude (β = 0.18^*^, ^*^p < 0.05); the higher the risk perception of tourists traveling abroad for vaccination, the higher their pandemic prevention attitude. On the contrary, tourism risk perception has no significant impact on travel decision making (β = −0.02, *p* > 0.05) or travel intention (β = 0.09, *p* > 0.05), and the risk perception of tourists traveling abroad for vaccination has no impact on their travel decision-making or travel intention.

Furthermore, there is a significant positive relationship between the pandemic prevention attitude (β = 0.30^*^, ^*^p < 0.05) and travel decision making (β = 0.17^*^, ^*^p < 0.05) of tourists and their intention to travel abroad for vaccination; the more positive the pandemic prevention attitude and travel decision making of tourists, the higher their intention to travel abroad for vaccination. Moreover, tourists' risk perception has a significant indirect impact on their travel intention through the pandemic prevention attitude, and the pandemic prevention attitude plays a complete intervening role between the two. In other words, only when the risk perception of tourists traveling abroad for vaccination effectively strengthens their pandemic prevention attitude will there be a significant impact on their vaccine travel intention.

## Discussion

This study used the SOR model as the framework to explore the relationship between the risk perception, pandemic prevention attitude, travel decision-making, and travel intention of vaccine tourists. The risk perception of tourists has a significant positive impact on their pandemic prevention attitude. Their pandemic prevention attitude significantly impacts their travel intention, and pandemic prevention attitude is a complete intervening variable between risk perception and travel intention. Therefore, the relationship between the risk perception, pandemic prevention attitude and travel intention of vaccine tourists corresponds to the SOR model mechanism proposed by Mehrabian and Russell (28). Due to the shortage of vaccines in Taiwan, vaccine tourists are stimulated by their risk perception, which positively affects their pandemic prevention attitude, and eventually leads to the intention to travel abroad for vaccination. Ram and Chand (53) argued that risk perception can improve people's attitudes toward safety. When the risk perception is higher, people's attitude toward safety will be more rigorous, and generate the behaviors for personal safety. Therefore, the tourists' higher risk perception of COVID-19 actually strengthens intention to vaccinate abroad to ensure their life security.

In addition, the findings of this study are consistent with the findings of Bae and Chang (36), meaning that vaccine tourists regard traveling abroad for vaccination as a health promotion behavior, which results in a reduction of the risk perception of tourists and has a positive impact on their travel attitude. The relationship between pandemic prevention attitude and travel intention is the same as the findings of Tseng et al. (4), meaning that the better the pandemic prevention attitude of vaccine tourists, the higher their intention to travel abroad for vaccination.

Tourists' attention to their life safety and their desire to travel exceed travel risk perception and decision making. This study found no significant relationship between the risk perception and travel intention of vaccine tourists, which is inconsistent with most previous research results (7, 37–39). There are two possible reasons to explain why vaccine tourists in Taiwan do not reduce their intention to travel abroad for vaccination or their decision to travel abroad, despite the risks of traveling abroad during COVID-19. First, there is a severe shortage of vaccines in Taiwan, and most people have not been vaccinated. However, to maintain their daily life, they have to take the risk of being infected without antibodies to go out to work and buy goods. Thus, they feel their lives are threatened and choose to go abroad for vaccination. Second, the impact of the long-term ban on travel caused by COVID-19 results in tourists' perceptions of the severity of the pandemic having no significant impact on their travel intention (54). Thus, people's desire to travel abroad continues to accumulate, and vaccine tourism maybe meets people's desire to travel abroad. Thus, in the short term, especially in countries with a shortage of vaccines, vaccine tourism can be regarded as a way to alleviate the shortage of vaccines and meet the desire to travel.

There is a significant relationship between vaccine tourists' travel decision-making and travel intention, corresponding to previous tourism-related research results (42–44). Vaccine tourists' confidence in the effectiveness of the government's pandemic prevention policies and the medical system of the destination are the main factors influencing tourists' intention to travel abroad for vaccination. Thus, the perceived value is transformed into the actual intention of tourism after receiving information about the destinations and conducting internal evaluation (42). The implementation of pandemic prevention policies and the supply of medical quality in the tourist destinations of vaccine tourism are the key factors that determine tourists' travel intention. Therefore, before implementing vaccine tourism, the pandemic prevention policies between the two countries must be consensus-based and thoroughly implemented. In addition, the local medical system must be sound enough to provide sufficient vaccinations and medical care for international tourists and meet the pandemic control measures in Taiwan.

### Limitations of the study

While this study was carried out with a rigorous design, there are still several limitations to be further studied:

1. The research scope of this study is limited to Taiwan, and other cases with the same shortage of vaccines are not included in the scope of this study; therefore, future studies can verify whether there are similar findings in cases with similar situations.
2. The relationship between risk perception and travel intention in cases with abundant vaccines is worth exploring and comparing.
3. This study conducted quantitative research. Thus, qualitative research can be added to the research design in the future to enhance the understanding of vaccine tourists' travel intentions.

## Conclusion

The risk perception of vaccine tourists has a significant impact on the pandemic prevention attitude, and the pandemic prevention attitude is a complete intervening variable between risk perception and travel intention. Due to the shortage of vaccines and a long-standing travel ban in Taiwan, tourists' risk perceptions do not have a significant impact on their travel intention or decision making. However, vaccine travel decision-making has a significant impact on tourists' travel intentions, which mainly depend on the local tourism destinations' pandemic prevention policy and medical quality. In the short term, as a product of COVID-19, vaccine tourism can alleviate the tourism economic dilemma for countries with sufficient medical supplies and ease the desire of tourists to travel. Therefore, the pandemic prevention planning and medical quality of the tourism destinations and the supportive measures in inbound and outbound destination must be carefully reviewed. The government can use direct and indirect management policy forcing vaccine tourists to obey the national pandemic prevention.

## Data Availability Statement

The raw data supporting the conclusions of this article will be made available by the authors, without undue reservation.

## Author Contributions

X-BW contributions in the study included methodology, conceptualization, and original draft. C-CC contributions in the study were investigation, methodology, and original draft. GK contributions in the study were formal analysis, methodology, and review and editing. C-HC contributions in the study were investigation, project administration, and resources. CH contributions in the study were supervision and review and editing. P-YL contributions in the study were data curation and project administration. All authors contributed to the article and approved the submitted version.

## Conflict of interest

The authors declare that the research was conducted in the absence of any commercial or financial relationships that could be construed as a potential conflict of interest.

## Publisher's note

All claims expressed in this article are solely those of the authors and do not necessarily represent those of their affiliated organizations, or those of the publisher, the editors and the reviewers. Any product that may be evaluated in this article, or claim that may be made by its manufacturer, is not guaranteed or endorsed by the publisher.
